# Comparative analysis and validation of the malachite green assay for the high throughput biochemical characterization of terpene synthases

**DOI:** 10.1016/j.mex.2014.08.007

**Published:** 2014-09-08

**Authors:** Maria Vardakou, Melissa Salmon, Juan A. Faraldos, Paul E. O’Maille

**Affiliations:** a Department of Metabolic Biology, John Innes Centre, Norwich Research Park, Norwich NR4 7UH, United Kingdom; b Food and Health Programme, Institute of Food Research, Norwich Research Park, Norwich NR4 7UA, United Kingdom; c School of Chemistry, Cardiff University, Main Building, Park Place, Cardiff CF10 3AT, United Kingdom

**Keywords:** Steady-state kinetics, Catalytic efficiency, Turnover number, Terpene synthases, Colorimetric assay, High-throughput, Screening, Malachite green, TPS, terpene synthase, MG, malachite green, FPP, farnesyl diphosphate, PPi, pyrophosphate, Pi, monophosphate

## Abstract

Terpenes are the largest group of natural products with important and diverse biological roles, while of tremendous economic value as fragrances, flavours and pharmaceutical agents. Class-I terpene synthases (TPSs), the dominant type of TPS enzymes, catalyze the conversion of prenyl diphosphates to often structurally diverse bioactive terpene hydrocarbons, and inorganic pyrophosphate (PPi). To measure their kinetic properties, current bio-analytical methods typically rely on the direct detection of hydrocarbon products by radioactivity measurements or gas chromatography–mass spectrometry (GC–MS).

In this study we employed an established, rapid colorimetric assay, the pyrophosphate/malachite green assay (MG), as an alternative means for the biochemical characterization of class I TPSs activity.

• We describe the adaptation of the MG assay for turnover and catalytic efficiency measurements of TPSs.
• We validate the method by direct comparison with established assays. The agreement of *k*_cat_/*K*_M_ among methods makes this adaptation optimal for rapid evaluation of TPSs.
• We demonstrate the application of the MG assay for the high-throughput screening of TPS gene libraries.

## Graphical abstract

**Figure:**
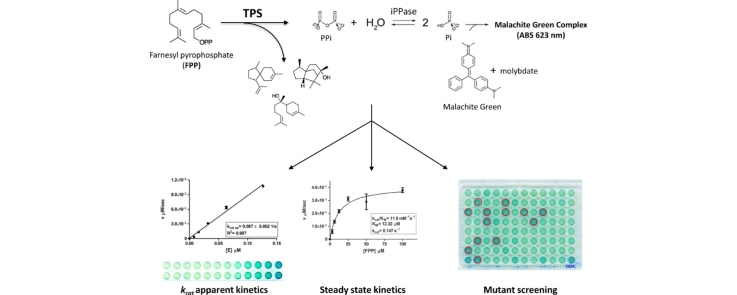

## Method details

### Substrate and reagents

Farnesyl diphosphate (FPP) was synthesized following the procedures developed by Meyers [1] and Poulter [2] starting from (2*E*,6*E*)-farnesol; malachite green powder; pure (*E*)-β-farnesene and all other reagents were purchased from commercial vendors.

### Protein expression and purification

To evaluate the MG/pyrophosphate assay as a tool for the biochemical characterization of TPSs, we chose three well studied enzymes as exemplars, namely *Artemisia annua* β-farnesene synthase (AaFS), *A. annua* amorpha-4,11-diene synthase (AaADS), and *Nicotiana tabacum* 5-epi-aristolochene synthase (TEAS). Gene constructs of AaFS, AaADS, and TEAS were inserted into the expression vector pH9GW (Gateway cloning system), and introduced into *Escherichia coli* BL21 (DE3) cells. Cell cultures were grown at 37 °C in Terrific Broth (TB) complemented with kanamycin (50 μg/mL). Protein expression was induced, at OD 600 nm ≥ 0.8, with 0.1 mM IPTG. The cultures were incubated with shaking for a further 5 h at 20 °C. Cells were harvested by centrifugation. Cell pellets were re-suspended in 50 mL of buffer A (50 mM Tris–HCl, 50 mM glycine, 5% glycerol, 0.5 M NaCl and 20 mM imidiazole, pH 8), complemented with an EDTA-free protease inhibitor tablet.

The cells were lysed (cell disruptor, 25 kpsi) and the clarified lysate was loaded onto a FPLC apparatus, AKTAxpress, for a two-step purification using a 5 mL Ni^2+^-immobilized metal ion affinity chromatography column (HisTrap™ HP), equilibrated with buffer A, and an S200 26/60 Sephadex Gel filtration column equilibrated with 20 mM HEPES, 0.15 M NaCl, at pH 7.5. Fractions containing purified protein were measured using the Bradford assay. Protein purity was verified with SDS-PAGE chromatography.

### Protein expression and purification (for library screening)

Mutant clones were transformed into BL21 (DE3) and spread onto LB plates with kanamycin. Individual colonies were transferred in 3 mL LB with kanamycin, in 24-well plates, and incubated o/n (37 °C, 230 rpm). 0.5 mL of each o/n culture was diluted to 5 mL with TB containing kanamycin. Growth was sustained at 37 °C in 24-well round bottom plates covered with micro-porous tape, until cultures reached OD600 ≥ 0.8. Protein expression was induced by addition of 0.1 mM IPTG at 20 °C. After 5 h, cells were harvested by centrifugation and stored at −20 °C until further use.

For the protein purification, pellets were re-suspended (25 °C) in 0.8 mL lysis buffer (50 mM Tris–HCl, 500 mM NaCl, 20 mM imidazole, 10% glycerol (v/v), 10 mM β-mercaptoethanol, and 1% (v/v) Tween-20, pH 8) containing lysozyme (1 mg/mL) and 1 mM EDTA for 30 min. Subsequently, 10 μL of benzonase solution (850 mM MgCl_2_ and 3.78 U/μL benzonase) was added with shaking at 250 rpm for 15 min.

The lysate (in 400 μL portions) was passed through a Whatman unifilter 96-well plate, and collected in another Whatman plate containing 50 μl bed-volume of superflow Ni-NTA resin, pre-equilibrated with lysis buffer using a vacuum manifold. Each well was washed with lysis buffer (3× 500 μL), followed by wash buffer (3× 500 μL, lysis buffer lacking Tween-20). After air drying, the resin was suspended in 150 μL elution buffer (wash buffer containing 250 mM imidazole) and centrifuged at 1500 rpm for 2 min. The eluate was reapplied to the column and the centrifugation step was repeated. Protein concentration was measured using the Bradford method.

### Radioactive enzyme assay

Kinetics assays were carried out according to the standard, linear range, micro-assay procedure developed for monoterpene synthases [3] with modifications [4]. This protocol involved incubation of varying amounts of [1-^3^H] FPP (specific activity 68 mCi/mmol) with a fixed concentration of purified enzyme (7.5 nM, 6.3 nM and 30.8 nM final concentration of AaFS, AaADS, and TEAS respectively) in 1× MTC buffer (25 mM MES; 25 mM CAPS; 50 mM Tris) containing 5 mM MgCl_2_, at pH 7.5. The enzymatic reactions were run in parallel, and the steady-state kinetic parameters obtained for each enzyme (AsFS, AaADS and TEAS) are the result of three independent runs.

### Enzyme vial assay and quantification by GC–MS

The vial assay was performed as previously described [5] in a 500 μL reaction volume using 2 mL screw-top GC glass vials. Each reaction consisted of assay buffer at pH 7.5 [25 mM 2-(N-morpholino)ethanesulfonic acid (MES), 25 mM N-cyclohexyl-3-aminopropanesulfonic acid (CAPS), 50 mM Tris(hydroxymethyl)aminomethane (Tris)], 5 mM MgCl_2_, FPP in concentration ranging from 0.78 to 200 μM and protein (0.014 μM final concentration unless otherwise stated).

Reaction products and (*E*)-β-farnesene standards were analyzed using a Hewlett–Packard 6890 gas chromatograph (GC) coupled to a 5973 mass selective detector (MSD) outfitted with a 7683B series injector and autosampler and equipped with an HP-5MS capillary column (0.25 mm i.d. × 30 m with 0.25 μm film). The (*E*)-β-farnesene peak was quantified by integration of peak areas using Enhanced Chemstation (version E.02.00), using a calibration curve from (*E*)-β-farnesene standards of different concentrations.

## Malachite green (MG) assay

### *k*_cat_ apparent determination

The MG assay was performed in 96-well flat bottomed plates in a total reaction volume of 50 μL consisting of malachite green assay buffer [25 mM MES, 25 mM CAPS, 50 mM Tris, 2.5 mU of the coupling enzyme inorganic pyrophosphatase (*Saccharomyces cerevisiae*), 5 mM MgCl_2_] at pH 7.5, serial dilutions of protein from 0.003 μM to 0.2 μM and a fixed concentration of farnesyl pyrophosphate FPP (100 μM). Standard curves (0.01–50 μM) of monophosphate (Pi) and pyrophosphate (PPi) were generated using serial 2-fold dilutions in malachite green assay buffer but without FPP. Reactions (in triplicate) were set up on ice and incubated at 30 °C for 15–40 min. Enzymatic reactions were quenched by addition of 12 μL of the malachite green development solution and incubated for 15 min prior readings at 623 nm on a Varioskan Flash plate reader. The malachite green development solution was prepared fresh before each experiment by mixing 10 mL of malachite green dye stock solution with 2.5 mL of 7.5% ammonium molybdate and 0.2 mL 11% Tween 20 [6]. According to our experience the development solution can be used for up to 5 hours after mixing. The malachite green dye stock solution was prepared according to Pegan et al. [6]. In short, 300 mL of 18 M sulfuric acid stock was mixed with 1.5 L of water and then cooled down to room temperature. Malachite green powder (2.2 g) was then added and mixed by stirring with a magnetic stir bar and stir plate.

**Fig. 1:**
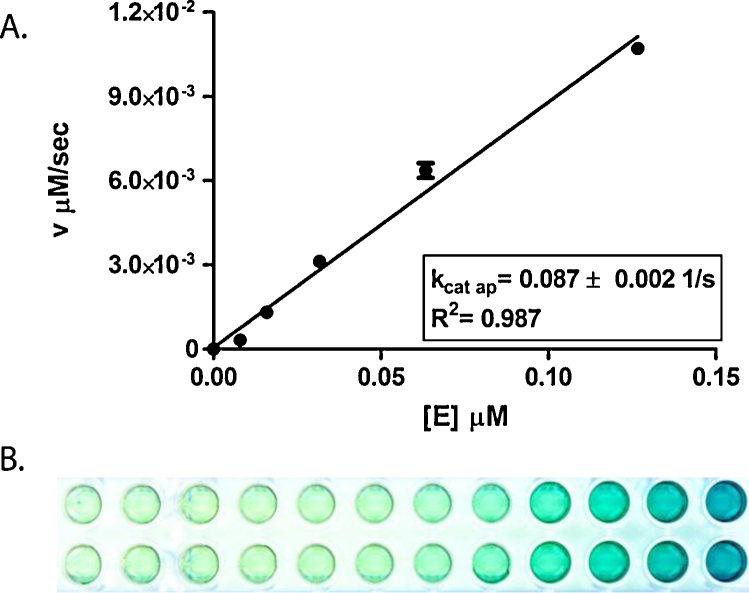
Linear range of enzymatic activity by the MG assay. (A) PPi produced (equivalent to consumed FPP) per sec vs. AaFS enzyme concentration. The reaction was terminated with the addition of Malachite Green solution after 15 min incubation at room temperature. The calculated slope after linear regression is equivalent to the *k*_cat_ apparent of the enzyme. (B) A typical colorimetric response generated 15 min following the addition of Malachite Green solution.

Linear regression analysis of the resulting plot (enzyme activity vs. enzyme concentration) enabled calculation of the apparent turnover number (*k*_cat ap_), which is equal to the slope of the line over the linear range of enzyme activity. The *k*_cat ap_ is a kinetic parameter convenient for identifying and optimizing the reaction conditions (temperature, pH, metal ion concentration, etc.) [7]. Importantly, this experiment precisely defines the kinetic conditions (linear range of enzyme activity) to be subsequently used in steady-state experiments. To validate the reproducibility of the MG assay in measuring the *k*_cat ap_ of the enzymatic reaction, we repeated identical assay measurements using several independent protein preparations of AaFS. In all cases the results were reproducible and the calculated *k*_cat ap_ was found to be between 0.075 and 0.090 s^−1^. A representative graph of these experiments is shown in Fig. 1 (*k*_cat ap_ = 0.087 ± 0.002, *R*^2^ = 0.987).

### Steady-state kinetic measurements

To validate the MG assay for steady-state kinetics, we conducted parallel experiments with the radioactive method using the same enzyme preparation of AaFS, AaADS, and TEAS. The radioactive assay was carried out as described above using a 0–10 μM concentration of [1-^3^H] FPP. Of note, the MG assay required a 10-fold increase in FPP concentrations to have a measurable spectrophotometric signal at comparable enzyme concentrations, but otherwise conducted under identical reaction conditions. Thus, the MG assay was performed starting at 100 μM FPP and subsequent serial dilutions and a constant enzyme concentration of 0.014 μM unless otherwise stated. Steady-state kinetic parameters (*K*_M_, *k*_cat_ and *k*_cat_/*K*_M_) were obtained after non-linear regression analysis of the data, using the Michaelis–Menten model, with GraphPad Prism. Results are summarized in Table 1.

**Table 1:** Steady-state kinetic parameters calculated from the malachite green assay (MG), the radioactive assay (RA) and the GC–MS vial assay.

Enzyme	AaFS			AaADS		TEAS	
	MG^a^	RA^b^	GC–MS^a^	MG^c^	RA^b^	MG^c^	RA^b^
*K*_M_ (μM)	12.3	1.9	11.9	8.9	2.0	23.3	3.4
*k*_cat_ (s^−1^)	0.147	0.020	0.198	0.030	0.006	0.030	0.004
[*k*_cat_/*K*_M_ (mM^−1^ s^−1^)]	11.9	10.3	16.6	3.4	3.1	1.3	1.2

a FPP concentration ranged from 0 to 100 μM.

b FPP concentration ranged from 0 to 10 μM; the *k*_cat_ and *K*_M_ measured with the radioactive assay were in agreement to those previous reports [33], [34], [35].

c FPP concentration ranged from 0 to 200 μM.

A head to head comparison between the MG and radioactive methods for all three enzymes reveals that both *k*_cat_ and *K*_M_ are relatively higher (4- to 7-fold) for the MG assay. However, the catalytic efficiency (*k*_cat_/*K*_M_) measured was found to be nearly identical for both assays (Table 1).

**Fig. 2:**
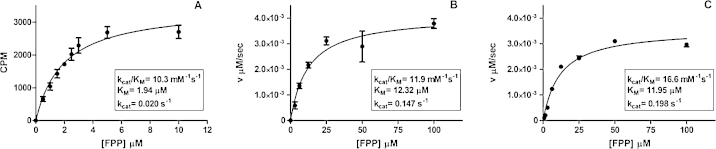
Michaelis–Menten kinetics of AaFS by the radioactive, the malachite green and the GC–MS vial assays. A non-linear regression analysis was performed on the data collected from the steady-state kinetic assays on AaFS. (A) Radioactive assay (RA; CPM vs. starting FPP concentration); (B) malachite green assay (MG; μM FPP catalyzed per sec vs. starting FPP concentration); and (C) GC–MS vial assay (GC–MS; μM FPP catalyzed per sec vs. starting FPP concentration).

As an additional comparison, we performed the GC–MS vial assay for AaFS. The GC–MS vial assay was conducted under identical conditions as the MG assay with the exception that the total reaction volume of the former was scaled to 500 μL and overlaid with a 500 μL of hexane to trap the enzymatic volatiles. Of note, we quenched vial assay reactions by adding a solution of 1 M KOH, 0.5 M EDTA prior to vortexing since enzyme activity was found to persist after hexane extraction. The extracted hexane layer was directly analyzed by GC–MS. The steady state kinetic results are summarized in Table 1. The kinetic parameters (*k*_cat_ and *K*_M_) values obtained by the GC–MS, despite being again approx. 7-fold inflated when compared to the radioactive assay, were almost identical to those obtained from MG protocol (Fig. 2), thus demonstrating that the catalytic efficiency of AaFS can be precisely determined by the MG assay. It is obvious that the MG and GC–MS assays give consistently higher values for both *K*_M_ and *k*_cat_, The estimated constants by MG and GC–MS assays are inflated relative to RA assay due to the higher substrate concentration needed to generate a detectable signal. Remarkably, when calculating the enzymes’ catalytic efficiencies (*k*_cat_/*K*_M_) the values across the different methods favourably compare to each other (Table 1). Taken together, our experimental results indicate that the MG assay is a viable method for partial biochemical characterizations of, and comparisons between TPS enzymes.

### High-throughput screening of gene libraries

Due to its fast development time and quick and easy set-up, the MG assay can be adapted for high-throughput screening of terpene synthase gene libraries synthesized via structure-based combinatorial protein engineering (SCOPE) [8]. For the simultaneous screening of large number of mutants, the MG assay is easily used in a 96-well format. For TPS enzymatic activity screenings, each mutant is expressed in a 5 mL culture and purified on a 96-well Ni-NTA plate. Screening reactions require purified protein at a concentration between 1 and 3 μM, therefore to facilitate high-throughput applications, sufficient protein must be produced on a 5 mL scale as determined from pilot studies. Protein concentration can also be measured in a 96-well plate format, using the Bradford microassay protocol. Each reaction (50 μL) requires 5 μL purified protein and a prenyl PP substrate at 100 μM. Reaction incubation time is variable, ranging from 30 min, to select for very high activity mutants, up to 4 h for less active mutants. MG activity can then be plotted against protein concentration to generate thresholds for mutant selection by comparing to positive and negative controls. Positive controls typically include a wild-type enzyme as well as known mutants that have high, medium and low activity; an empty expression vector is used as a negative control to provide a minimum threshold for protein concentration and enzyme activity.

**Fig. 3:**
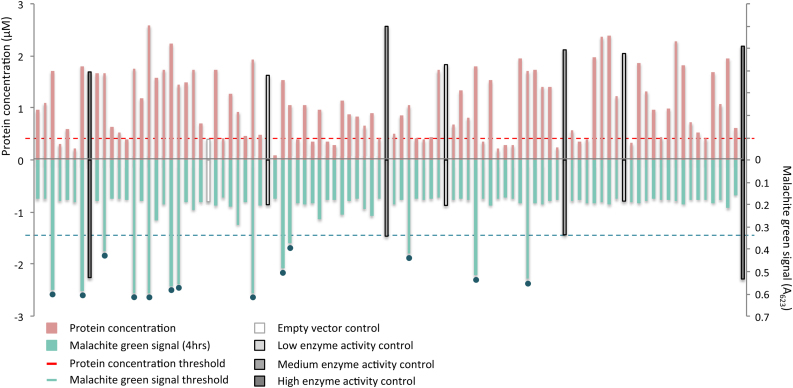
Comparative plot of protein concentration against malachite green signal for 96 TPS proteins from gene library screening. Eight controls consisting of the empty vector and low, medium and high activity controls are shown in shades of grey. The empty vector control provides the threshold for protein concentration whilst the medium activity enzyme controls provide a threshold for malachite green signal. These two thresholds can be used to select for high activity proteins that warrant further characterization.

This screening technique has been used to select highly active mutants from a gene library made in the background of *A. annua* (*E*)-β-farnesene synthase. To this end, 88 mutants were purified and assayed together with 8 controls (Fig. 3) to select for high activity mutants. Using the medium activity controls as a MG signal threshold, 13 mutants were selected. These mutants were further characterized by GC–MS analysis for product identification, and DNA sequencing to identify the location of the mutations.

The present study demonstrates the successful adaptation of the MG assay as an alternative enzymological method for the characterization of TPSs. The sensitivity of the MG assay is, for the most part, within the linear range of FPP concentrations used under steady-state conditions and hence, viable for the kinetic characterization of TPSs. The MG assay can be used to determine catalytic efficiencies (*k*_cat_/*K*_M_) with remarkable precision, as exemplified here using three mechanistically distinct sesquiterpene synthases. Using the MG assay for any given class I terpene synthase is also extremely useful for rapid identification of the optimum conditions of the enzyme (pH, temperature, presence of different metals, etc.). Furthermore, for highly expressed proteins, a 5 mL culture provides sufficient protein to reliably measure *k*_cat ap_ enabling the high-throughput kinetic analysis of libraries of mutant enzymes. Also, the MG assay can be easily adapted as a screening tool to identify active TPS enzymes that might require a more detailed biochemical analysis.

The MG assay is complementary to other methods, particularly the GC–MS that is essential for characterizing the product spectrum of native and mutant enzymes. As opposed to the radioactivity assay and GC–MS protocols, the MG assay benefits from requiring only minimum laboratory skills and equipment, while generating only standard bio- degradable/hazard waste (Table 2). While the current set-up of the assay is in a 96-well plate format, we believe the MG assay can be easily scaled to 384-well plates to measure the kinetic properties of many different protein preparations or mutants simultaneously. In addition, since the MG assay exclusively utilizes the released PPi co-product of class I TPS and numerous other isoprenoid biosynthetic enzymes (i.e. farnesyl diphosphate synthase), this methodology is not limited by the hydrocarbon product outcome or the chemical reaction mechanism of the TPS [9]. In summary, the MG assay is a very powerful bio-analytical tool that enables for the first time a quick, easy to handle, and cost effective measurement of the catalytic efficiency of class I terpene synthases.

**Table 2:** Comparison of TPSs enzyme assays.

	Radioactive assay	GC–MS vial assay	Malachite green assay
Detection limit^a^	>0.05 μM FPP	∼1.5 μM FPP	∼3 μM FPP
Run time^b^	4–5 h	8–10 h	<1 h
Throughput	Low-medium	Low	Medium-high
Cost	High	Medium	Low
Skills/expertise required	Medium-high	Medium	Low
Optimal use	Calculation of steady-state parameters	Product identification	Calculation of catalytic efficiency, activity screening, characterization of optimum pH, Temperature, etc.

a Expressed as the lowest usable FPP concentration in assay's reaction mixture.

b Run time for the completion one steady-state experiment (single protein; 8–10 substrate concentrations) in triplicate.

## Additional information

### Background

Terpene synthases (TPSs) catalyze the transformation of linear, achiral isoprenoid diphosphates into structurally diverse, often polycyclic and stereochemically complex terpene hydrocarbons. Class-I TPSs, the dominant type of TPS enzymes, are metal-dependent carbon–oxygen lyases (EC 4.2.3) which operate by cleaving the C—O σ-bond of the pyrophosphate ester functionality of the substrate to generate a carbocation that can then undergo a series of electrophilic cyclizations and rearrangements. The enzymatic reaction (or catalytic cycle), is terminated by carbocation neutralization via proton loss or addition of a nucleophile to produce a hydrocarbon and a molecule of inorganic pyrophosphate [10]. Hundreds of cyclic monoterpene (C10) [11], sesquiterpene (C15) [12], and diterpene (C20) [13], [14] carbon skeletons result from the precise carbocationic-guided transformations (reaction cascades) mediated by the individual TPSs. Myriad downstream enzymes (cytochrome p450's, glycosyltransferases, acyltransferases, etc.) subsequently tailor terpene hydrocarbon scaffolds to produce the wealth of terpenoids found in nature.

An increasing body of sequence information from bioinformatics analysis of genomics data indicates that an enormous number of terpene synthases are encoded by plants and microorganisms with several hundred in bacteria alone [15]. These observations coupled with the biological and industrial importance of terpenes and TPSs, have recently motivated the development of novel biochemical methods for kinetic characterization of TPSs [5], discovery of TPS enzymatic activity [9], and chemical characterization of TPS enzymatic products [15]. While gas chromatography–mass spectrometry (GC–MS) has been the principle method for the analysis of volatiles produced by TPSs, the radioactive assay has been the bio-analytical tool of choice to assay the kinetic competency (or catalytic efficiency) of these enzymes. More recently, a GC–MS single vial assay was developed for product (sesquiterpenes) identification and adapted to enable enzyme characterization of libraries of mutant enzymes [7] and native plant TPSs [16] by standard steady-state kinetics [5], [17] with non-radioactive material. Steady-state kinetics of sesquiterpene synthases has on occasion been determined by measuring the release of pyrophosphate (PPi) in a commercially available fluorometric assay kit (PiPer™, Invitrogen). This later method however has not been, to our knowledge, validated against either of the well-established techniques (radioactive or GC–MS assay).

In this work, we described a rapid colorimetric assay for measuring the catalytic efficiency of TPSs and its applicability as a high-throughput screening tool of TPSs activity in vitro. We adapted the malachite green (MG) assay as a fast, accurate, and inexpensive alternative to existing methods for measuring kinetics of phosphate-producing enzymes. The MG assay has been used for a number of years as a simple colorimetric method for the determination of inorganic phosphate [18], [19], [20], [21], [22], [23], [24], [25] as well as for kinetic analysis and evaluation of enzymes involved in primary metabolism [26], [27], [28], [29], [30], [31]. On one occasion, it has been used for estimating optimal conditions for the activity of a prenyl diphosphate synthase [32]. In our study we employed the MG assay as a coupled assay wherein the pyrophosphate generated by a TPS is subsequently broken down by a pyrophosphatase to produce two molecules of monophosphate that further react with molybdenum to form a coloured complex that can be then measured by standard UV spectrometers. We demonstrated the utility of the MG assay by measuring the catalytic efficiency of three mechanistically distinct native enzymes from *A. annua* and *N. tabacum*. We validated the method by comparing the values estimated from the MG assay and the established radioactive and GC–MS vial assay on the same enzyme preparations. Finally, we demonstrated its potential as a tool for high-throughput applications.
